# Supplementary material for: Metabolic tagging of extracellular vesicles and development of enhanced extracellular vesicle based cancer vaccines
Source: Nat Commun. 2023 Dec 5;14:8047. doi: 10.1038/s41467-023-43914-8 (PMC10697976; doi:10.1038/s41467-023-43914-8)
Supplement: Supplementary file 1 — Supplementary Information [file 41467_2023_43914_MOESM1_ESM.pdf]

**Supplementary Figures for**

# Metabolic Tagging of Extracellular Vesicles and Development of Enhanced Extracellular Vesicle Based Cancer Vaccines

Rimsha Bhatta[1], JoonSu Han[1], Yusheng Liu[1], Yang Bo[1], David Lee[1], Jiadiao Zhou[1], Yueji Wang[1,2], Erik Russell Nelson[3,4,5,6], Qian Chen[1,4,7], Xiaojia Shelly Zhang[2,8,9], Wael Hassaneen[10,11], Hua Wang[1,3,4,5,7,10,12]*

[1]Department of Materials Science and Engineering, University of Illinois at Urbana-Champaign, Urbana, IL 61801, USA. [2]Department of Mechanical Science and Engineering, University of Illinois Urbana-Champaign, Urbana, IL 61801, USA. [3]Cancer Center at Illinois (CCIL), Urbana, IL 61801, USA. [4]Beckman Institute for Advanced Science and Technology, University of Illinois at Urbana-Champaign, Urbana, IL 61801, USA. [5]Institute for Genomic Biology, University of Illinois at Urbana-Champaign, Urbana, IL 61801, USA. [6]Department of Molecular and Integrative Physiology, University of Illinois at Urbana-Champaign, Illinois, USA. [7]Materials Research Laboratory, University of Illinois at Urbana-Champaign, Urbana, IL 61801, USA. [8]Department of Civil and Environmental Engineering, University of Illinois at Urbana-Champaign, Urbana, IL 61801, USA. [9]National Center for Supercomputing Applications, Urbana, IL 61801, USA. [10]Carle College of Medicine, University of Illinois at Urbana-Champaign, Urbana, IL 61801, USA. [11]Carle Foundation Hospital, Urbana, IL 61801, USA. [12]Department of Bioengineering, University of Illinois at Urbana-Champaign, Urbana, IL 61801, USA.

*correspondence should be addressed to huawang3@illinois.edu

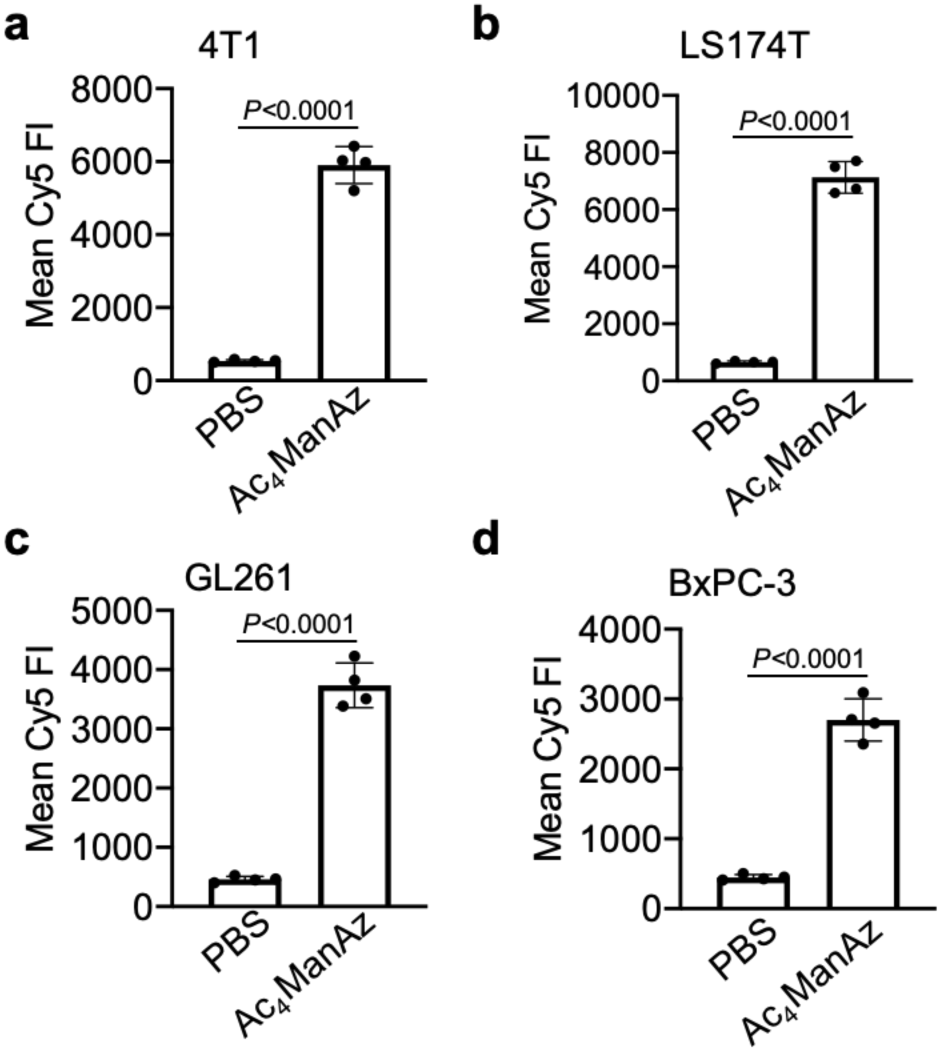


**Figure S1. $Ac_4ManAz$ can metabolically label cancer cells with azido groups.** Mean Cy5 fluorescence intensity of (a) 4T1 cells, (b) LS174T cells, (c) GL261 cells, and (d) BxPC-3 cells, respectively, after 3-day incubation with $Ac_4ManAz$ and 30-min incubation with DBCO-Cy5 (n=4). Cells treated with PBS and incubated with DBCO-Cy5 were used as controls. All the numerical data are presented as mean ± SD ($0.01 < {*}P \leq 0.05$; $**P \leq 0.01$; $***P \leq 0.001$; $****P \leq 0.0001$).

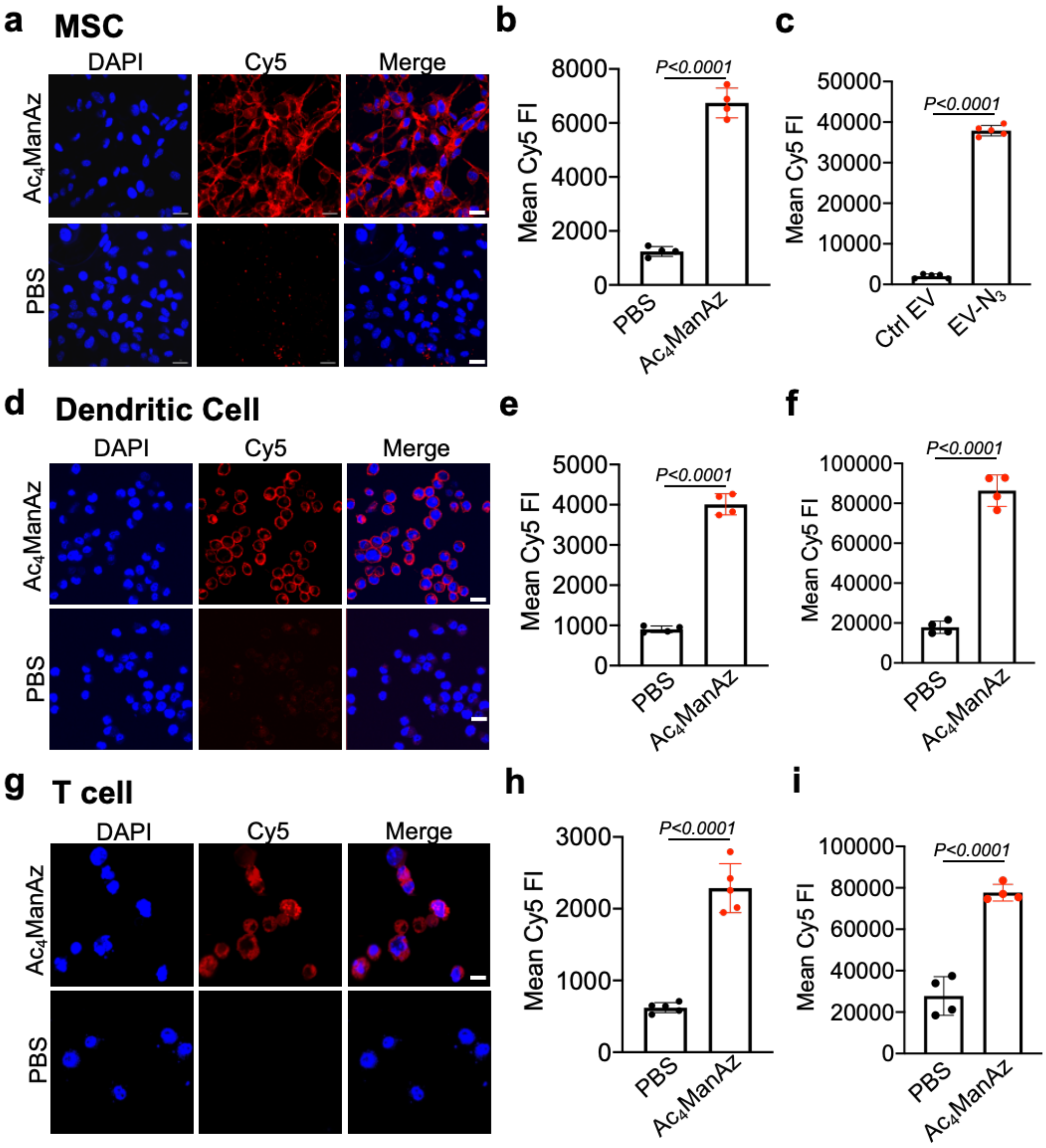


**Figure S2**. **The EV tagging approach is applicable to MSC, dendritic cells, and T cells**. (**a**) CLSM image and (**b**) Mean Cy5 fluorescence intensity (FI) of murine MSCs (n=4) after treated with $Ac_4ManAz$ for three days and incubated with DBCO-Cy5 (red) for 30 min. Cell nuclei were stained with DAPI (blue). Scale bar: 10 µm. (**c**) Mean Cy5 FI of EVs that were harvested from $Ac_4ManAz$-treated or untreated MSCs and stained with DBCO-Cy5 (n=5). (**d**) CLSM image and (**e**) Mean Cy5 FI of dendritic cells after treated with $Ac_4ManAz$ for three days and incubated with DBCO-Cy5 for 30 min (n=4). Scale bar: 10 µm. (**f**) Mean Cy5 FI of EVs that were harvested from $Ac_4ManAz$-treated or untreated dendritic cells and stained with DBCO-Cy5 (n=4). (**g**) CLSM image and (**h**) Mean Cy5 FI of T cells after treated with $Ac_4ManAz$ for three days and incubated with DBCO-Cy5 for 30 min (n=5). Scale bar: 10 µm. (**i**) Mean Cy5 FI of EVs that were harvested from $Ac_4ManAz$-treated or untreated T cells and stained with DBCO-Cy5 (n=4). All the numerical data are presented as mean ± SD ($0.01 < {*}P \leq 0.05$; $**P \leq 0.01$; $***P \leq 0.001$; $****P \leq 0.0001$).

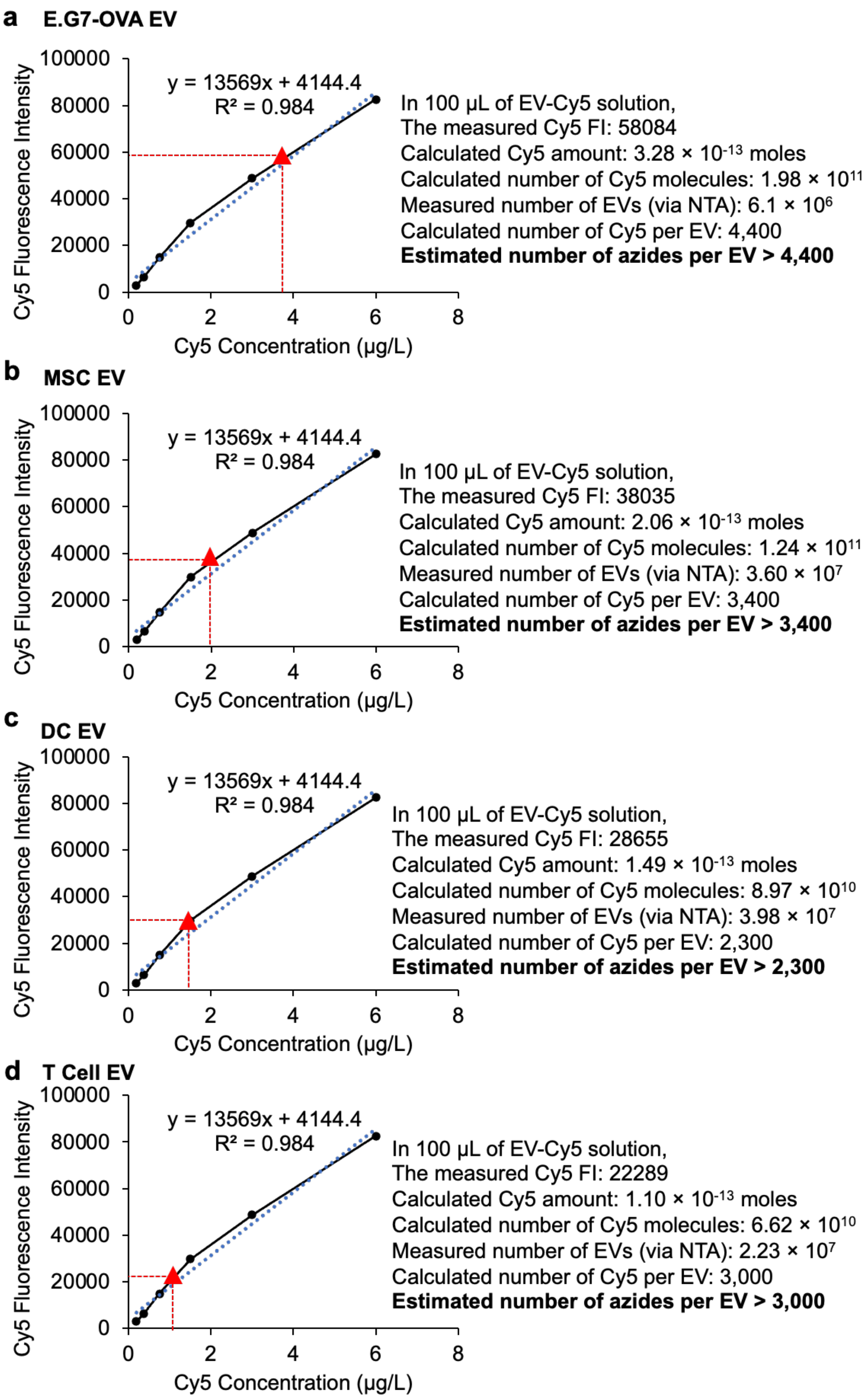


**Figure S3. Quantification of surface azido groups per EV**. Exosomes were collected from $Ac_4ManAz$- or PBS-treated cells and incubated with DBCO-Cy5 for 30 min. A standard curve of Cy5 fluorescence intensity was used to calculate the amount of Cy5 molecules that were conjugated to EVs derived from $Ac_4ManAz$-treated (a) E.G7-OVA cells, (b) MSCs, (c) DCs, and (d) T cells, respectively, as a means to estimate the number of azido groups per EV.

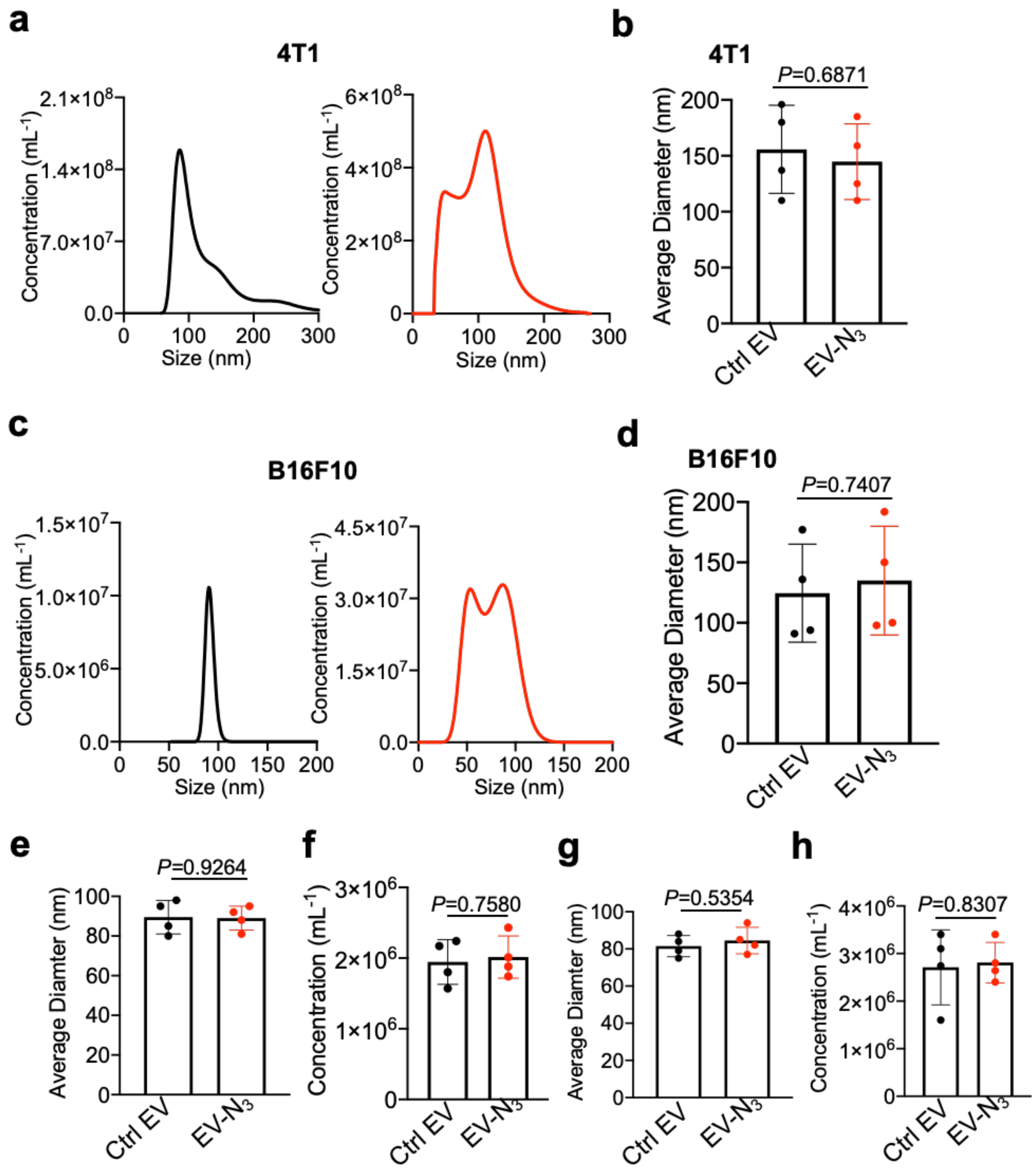


**Figure S4. Metabolic glycan labeling shows a minimal effect on EV secretion process of cancer cells**. (a) Representative size distribution of EVs isolated from untreated (black curve) or $Ac_4ManAz$-treated (red curve) 4T1 cells. (b) Average diameter of EVs isolated from untreated or $Ac_4ManAz$-treated 4T1 cells (n=4). (c) Representative size distribution of EVs isolated from untreated (black curve) or $Ac_4ManAz$-treated (red curve) B16F10 cells. (d) Average diameter of EVs isolated from untreated or $Ac_4ManAz$-treated B16F10 cells (n=4). (e) Average diameter and (f) concentration of EVs from untreated or $Ac_4ManAz$-treated DCs (n=4). (g) Average diameter and (h) concentration of EVs from untreated or $Ac_4ManAz$-treated T cells (n=4). All the numerical data are presented as mean ± SD ($0.01 < *P \leq 0.05$; $**P \leq 0.01$; $***P \leq 0.001$; $****P \leq 0.0001$).

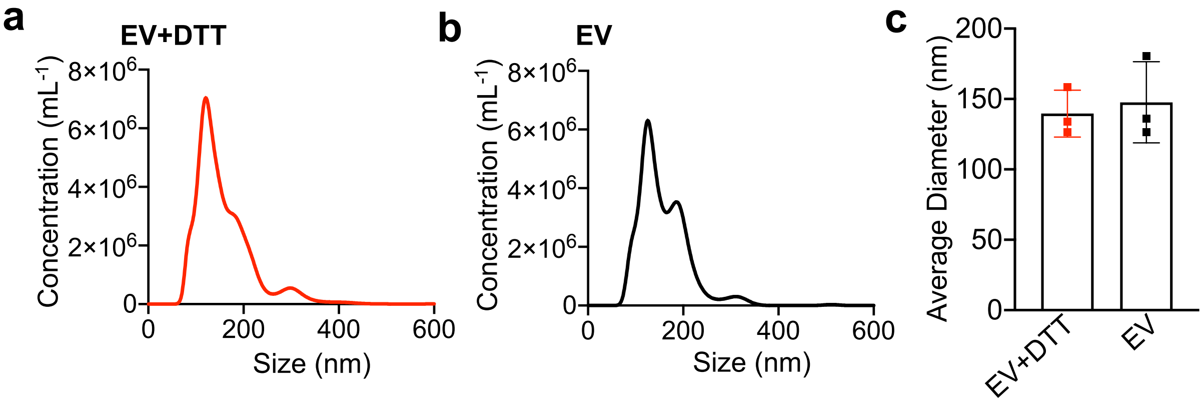


**Figure S5.** Representative size distribution of 4T1-derived EVs treated with (a) 10 mM DTT or (b) PBS for 10 minutes at room temperature. (c) Average diameter of EVs treated with DTT or PBS for 10 minutes (n=3).

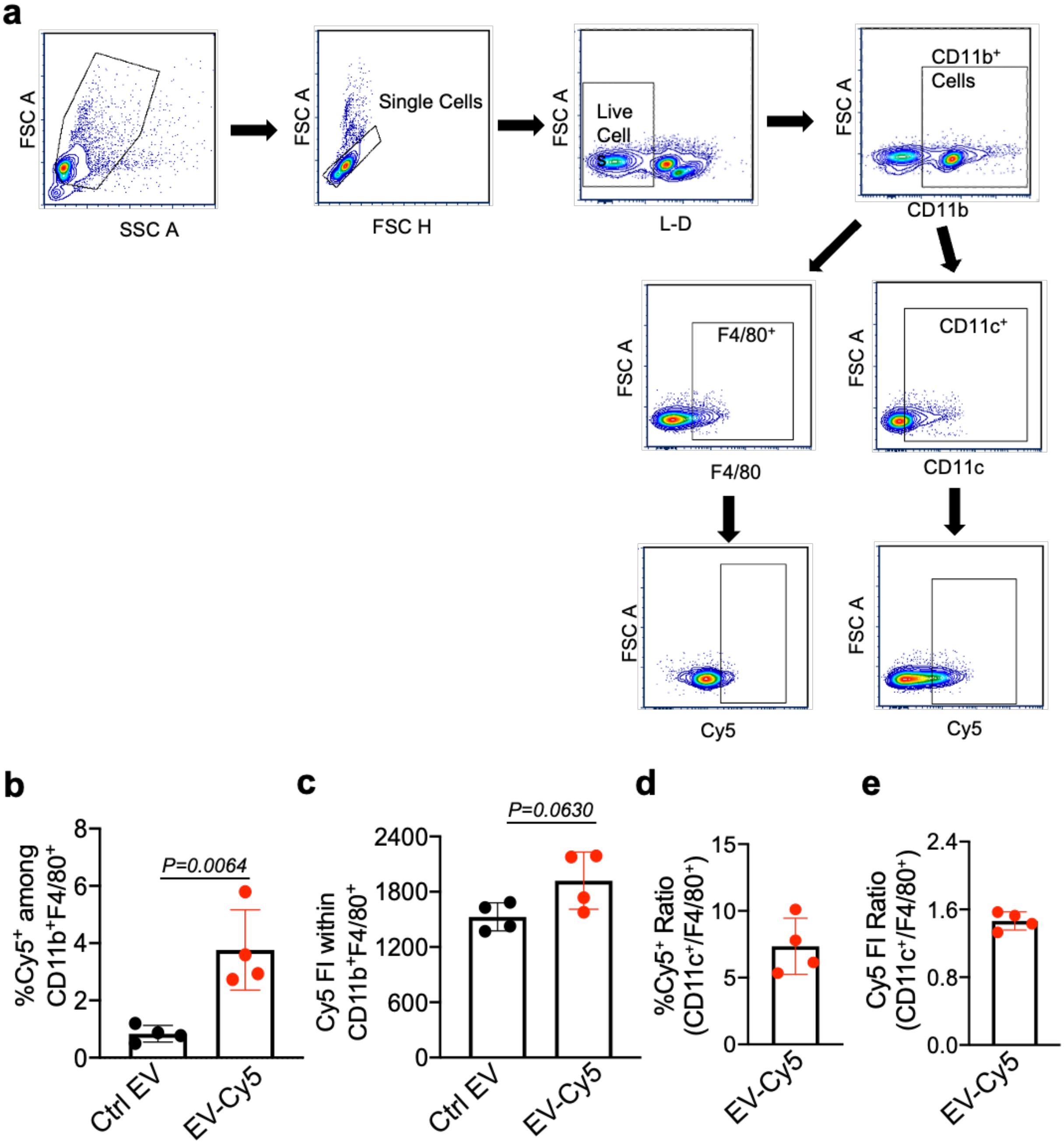


**Figure S6. *In vivo* tracking of subcutaneously injected Cy5-conjugated EVs**. Cy5-conjugated E.G7-OVA EVs or control EVs were subcutaneously injected into the flank of C57BL/6 mice, followed by FACS analysis of immune cells in the lymph nodes 16 hours later. (a) Representative gating strategy for analyzing the internalization of Cy5-conjugated EVs by immune cells in the draining lymph nodes. (b) Percentages of $Cy5^+$ macrophages ($CD11b^+F4/80^+$) (n=4). (c) Mean Cy5 fluorescence intensity of $CD11b^+F4/80^+$ macrophages (n=4). (d) Number ratio of $Cy5^+$ DCs to $Cy5^+$ macrophages (n=4). (e) Mean Cy5 FI ratio of $Cy5^+$ DCs to $Cy5^+$ Macrophages (n=4). All the numerical data are presented as mean ± SD ($0.01 < *P \leq 0.05$; $**P \leq 0.01$; $***P \leq 0.001$; $****P \leq 0.0001$).

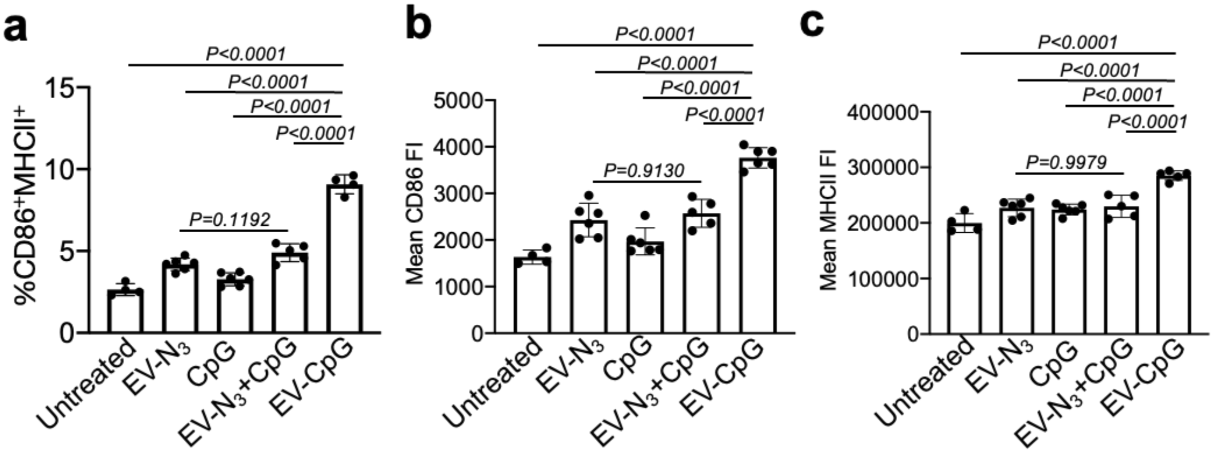


**Figure S7. CpG-conjugated B16F10 EVs result in improved activation of DCs.** DCs were treated with CpG-conjugated EVs, CpG, EVs, or the mixture of EVs and CpG for 16 h. The concentration of CpG and EVs was set at 1 nM and $1 \times 10^7$/mL, respectively. (a) Percentages of $CD86^+$ $MHCII^+$ DCs after different treatments (n=6). (b) Mean CD86 FI of DCs after different treatments (n=6). (c) Mean MHCII FI of DCs after different treatments (n=6). All the numerical data are presented as mean ± SD ($0.01 < *P \leq 0.05$; $**P \leq 0.01$; $***P \leq 0.001$; $****P \leq 0.0001$).

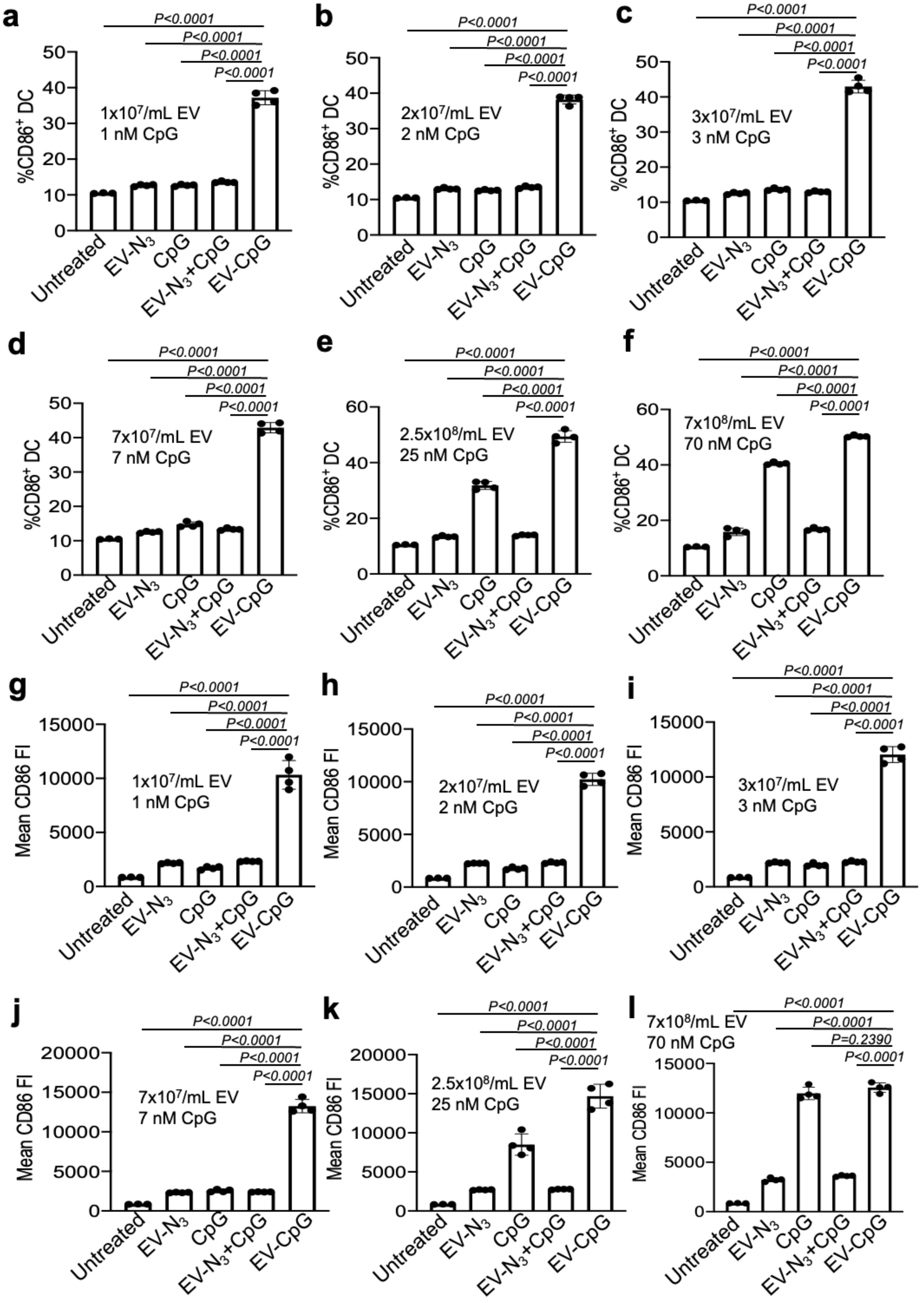

a
P<0.0001
P<0.0001
P<0.0001
P<0.0001
%CD86+ DC
1x10^7/mL EV
1 nM CpG
Untreated
EV-N3
CpG
EV-N3+CpG
EV-CpG
b
P<0.0001
P<0.0001
P<0.0001
P<0.0001
%CD86+ DC
2x10^7/mL EV
2 nM CpG
c
P<0.0001
P<0.0001
P<0.0001
P<0.0001
%CD86+ DC
3x10^7/mL EV
3 nM CpG
d
P<0.0001
P<0.0001
P<0.0001
P<0.0001
%CD86+ DC
7x10^7/mL EV
7 nM CpG
e
P<0.0001
P<0.0001
P<0.0001
P<0.0001
%CD86+ DC
2.5x10^8/mL EV
25 nM CpG
f
P<0.0001
P<0.0001
P<0.0001
P<0.0001
%CD86+ DC
7x10^8/mL EV
70 nM CpG
g
P<0.0001
P<0.0001
P<0.0001
P<0.0001
Mean CD86 FI
1x10^7/mL EV
1 nM CpG
h
P<0.0001
P<0.0001
P<0.0001
P<0.0001
Mean CD86 FI
2x10^7/mL EV
2 nM CpG
i
P<0.0001
P<0.0001
P<0.0001
P<0.0001
Mean CD86 FI
3x10^7/mL EV
3 nM CpG
j
P<0.0001
P<0.0001
P<0.0001
P<0.0001
Mean CD86 FI
7x10^7/mL EV
7 nM CpG
k
P<0.0001
P<0.0001
P<0.0001
P<0.0001
Mean CD86 FI
2.5x10^8/mL EV
25 nM CpG
l
7x10^8/mL EV
70 nM CpG
P<0.0001
P<0.0001
P=0.2390
P<0.0001
Mean CD86 FI

**Figure S8. CpG-conjugated tumor EVs upregulate surface expression of CD86 on DCs.** CpG-conjugated E.G7-OVA-derived EVs resulted in enhanced activation of DCs at different doses of EVs compared to EV alone, CpG alone, or the mixture of CpG and EVs. Shown are the percentages of $CD86^+$ DCs and mean CD86 FI of DCs after treatment with different groups at varied EV/CpG doses for 16 h: (a,g) $1\times10^7$/mL EVs and 1 nM CpG (n=4), (b,h) $2\times10^7$/mL EVs and 2 nM CpG (n=4), (c,i) $3\times10^7$/mL EVs and 3 nM CpG (n=4), (d,j) $7\times10^7$/mL EVs and 7 nM CpG (n=4), (e,k) $2.5\times10^8$/mL EVs and 25 nM CpG (n=4), or (f,l) $7\times10^8$/mL EVs and 70 nM CpG (n=4). All the numerical data are presented as mean ± SD ($0.01 < *P \leq 0.05$; $**P \leq 0.01$; $***P \leq 0.001$; $****P \leq 0.0001$).

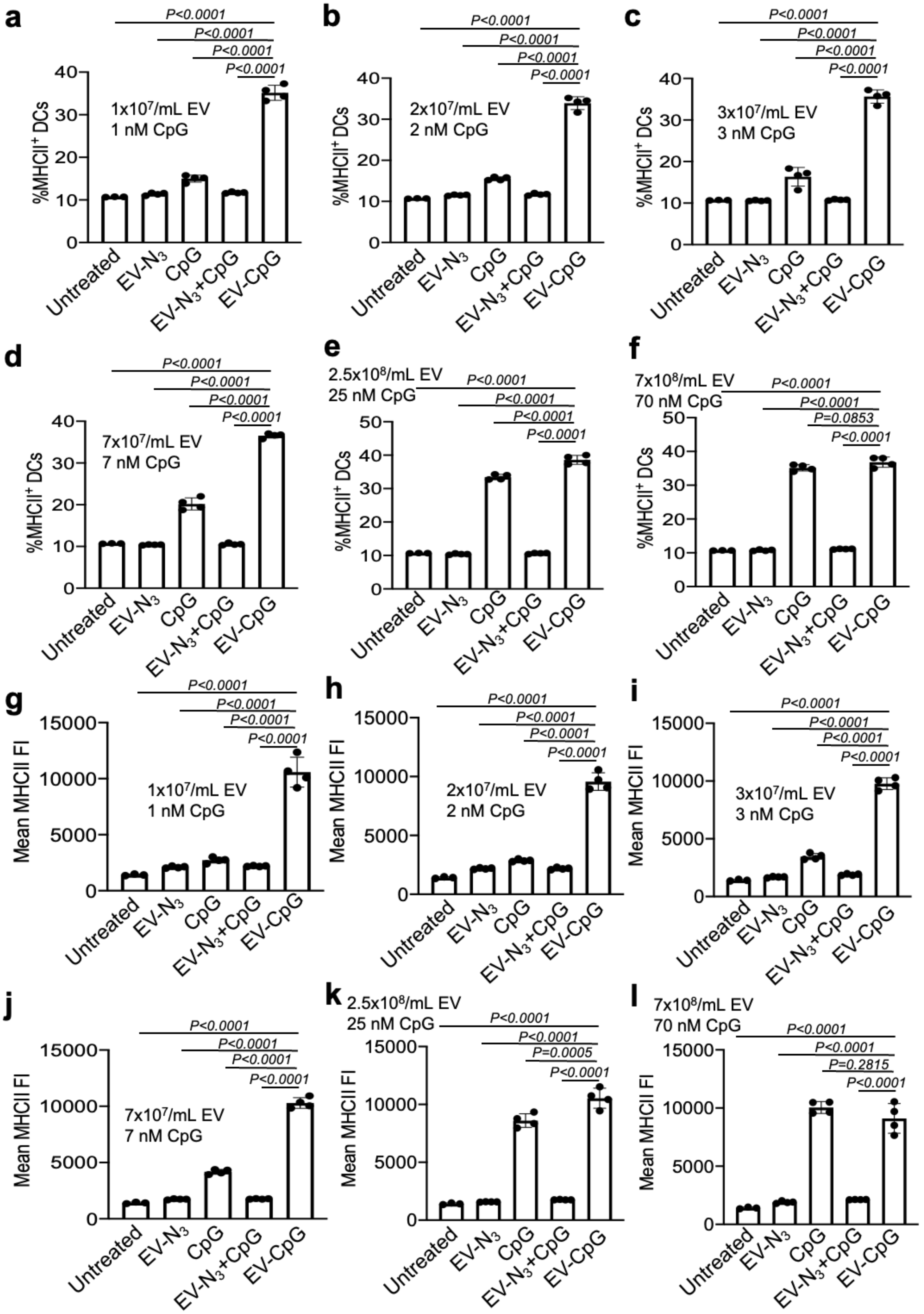
a
1x10^7/mL EV
1 nM CpG
%MHCII+ DCs
P<0.0001
Untreated
EV-N3
CpG
EV-N3+CpG
EV-CpG
b
2x10^7/mL EV
2 nM CpG
c
3x10^7/mL EV
3 nM CpG
d
7x10^7/mL EV
7 nM CpG
e
2.5x10^8/mL EV
25 nM CpG
f
7x10^8/mL EV
70 nM CpG
P=0.0853
g
Mean MHCII FI
h
i
j
k
P=0.0005
l
P=0.2815

**Figure S9. CpG-conjugated tumor EVs upregulate surface expression of MHCII on DCs.** CpG-conjugated E.G7-OVA-derived EVs resulted in enhanced activation of DCs at different doses of EVs compared to EV alone, CpG alone, or the mixture of CpG and EVs. Shown are the percentages of $MHCII^{+}$ DCs and mean MHCII FI of DCs after treatment with different groups at varied EV doses for 16 h: (a,g) $1\times10^{7}$/mL EVs and 1 nM CpG (n=4), (b,h) $2\times10^{7}$/mL EVs and 2 nM CpG (n=4), (c,i) $3\times10^{7}$/mL EVs and 3 nM CpG (n=4), (d,j) $7\times10^{7}$/mL EVs and 7 nM CpG (n=4), (e,k) $2.5\times10^{8}$/mL EVs and 25 nM CpG (n=4), or (f,l) $7\times10^{8}$/mL EVs and 70 nM CpG (n=4). All the numerical data are presented as mean ± SD ($0.01 < *P \leq 0.05$; $**P \leq 0.01$; $***P \leq 0.001$; $****P \leq 0.0001$).

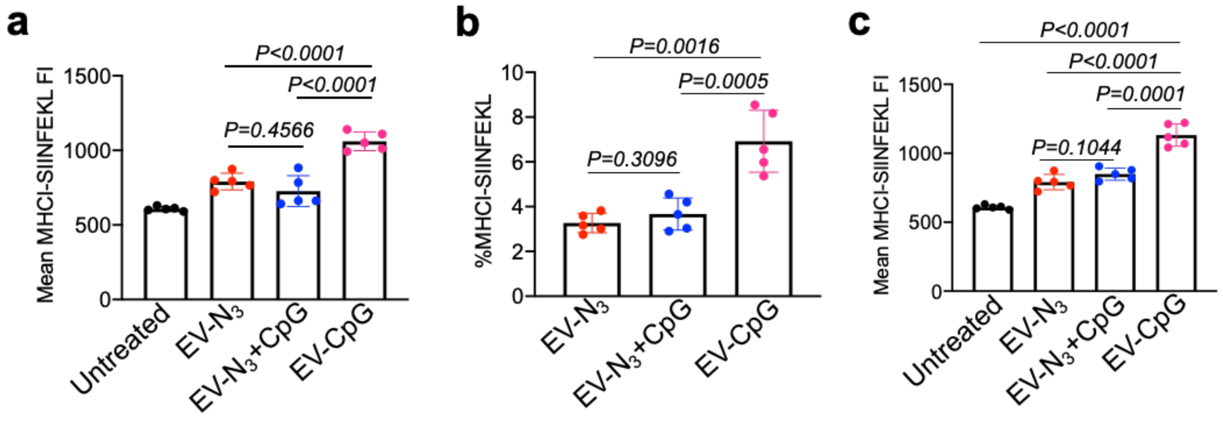


**Figure S10. CpG-conjugated E.G7-OVA EVs induce improved SIINFEKL presentation by DCs.** BMDCs were incubated with CpG-conjugated E.G7-OVA EVs, EV alone, or the mixture of CpG and EVs for 16 h. The concentrations of CpG and EVs were set at 1 nM and $1x10^7$/mL, respectively for (a) and 5 nM and $1x10^7$/mL, respectively for (b-c) (n=5). (a) Mean MHCI-SIINFEKL fluorescence intensity of DCs after different treatments. (b) Percentages of MHCI-SIINFEKL$^+$ DCs and (c) mean MHCI-SIINFEKL fluorescence intensity of DCs after different treatments. All the numerical data are presented as mean ± SD ($0.01 < *P \leq 0.05$; $**P \leq 0.01$; $***P \leq 0.001$; $****P \leq 0.0001$).

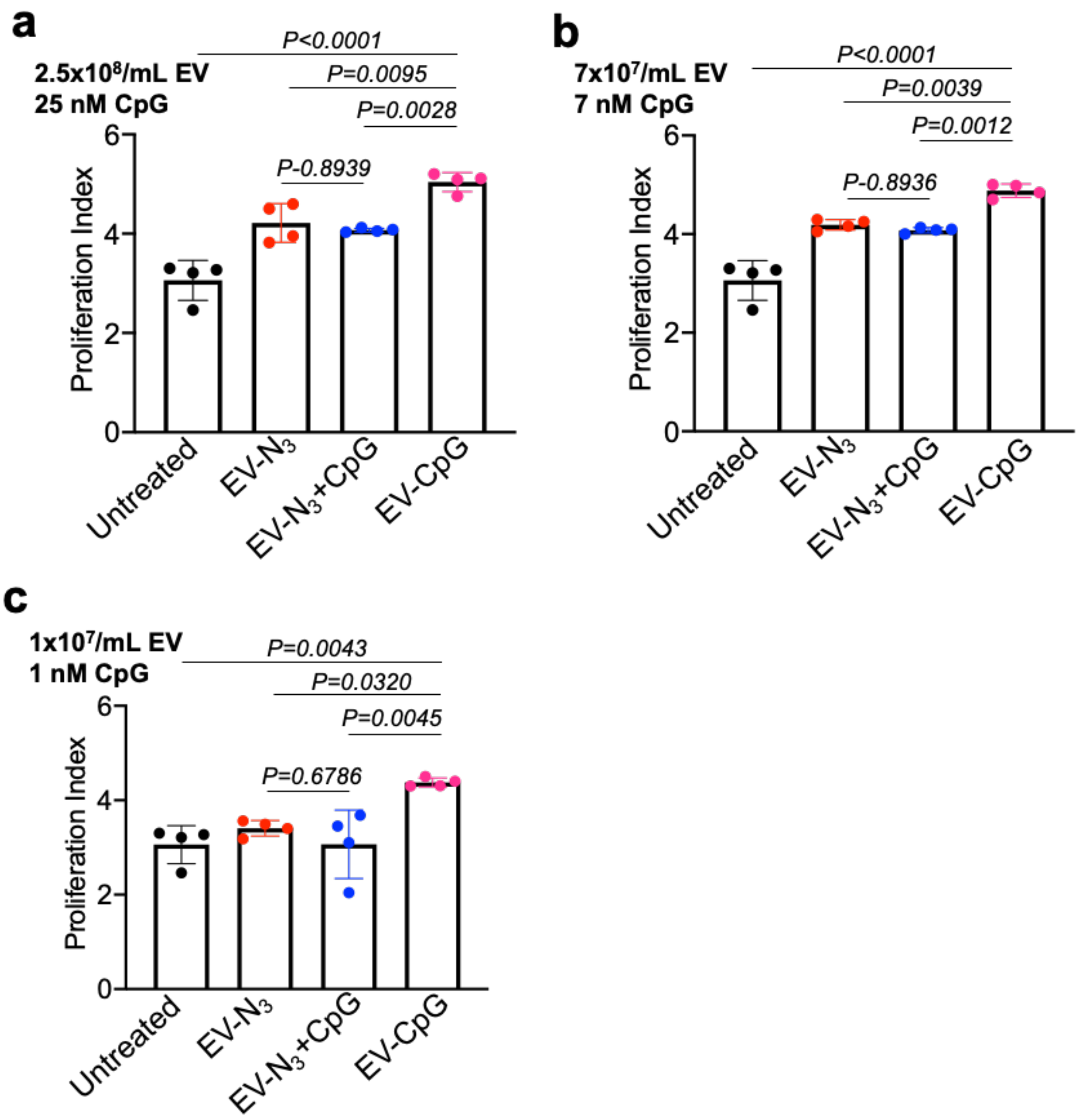


**Figure S11. DCs treated with CpG-conjugated E.G7-OVA EVs result in enhanced proliferation of SIINFEKL-specific OT-1 cells.** DCs were pretreated with CpG-conjugated EVs, EV alone, the mixture of CpG and EVs, or PBS (n=4). The concentration of exosomes and CpG were set at $2.5 \times 10^8$/mL and 25 nM for (a), $7 \times 10^7$/mL and 7 nM for (b), and $1 \times 10^7$/mL and 1 nM for (c). DCs were then co-incubated with CFSE-stained OT-1 cells for three days, followed by FACS analysis of OT-1 cell proliferation. Shown are the proliferation index of OT-1 cells after 3-day incubation with DCs. All the numerical data are presented as mean ± SD ($0.01 < {}^{*}P \leq 0.05$; $^{**}P \leq 0.01$; $^{***}P \leq 0.001$; $^{****}P \leq 0.0001$).

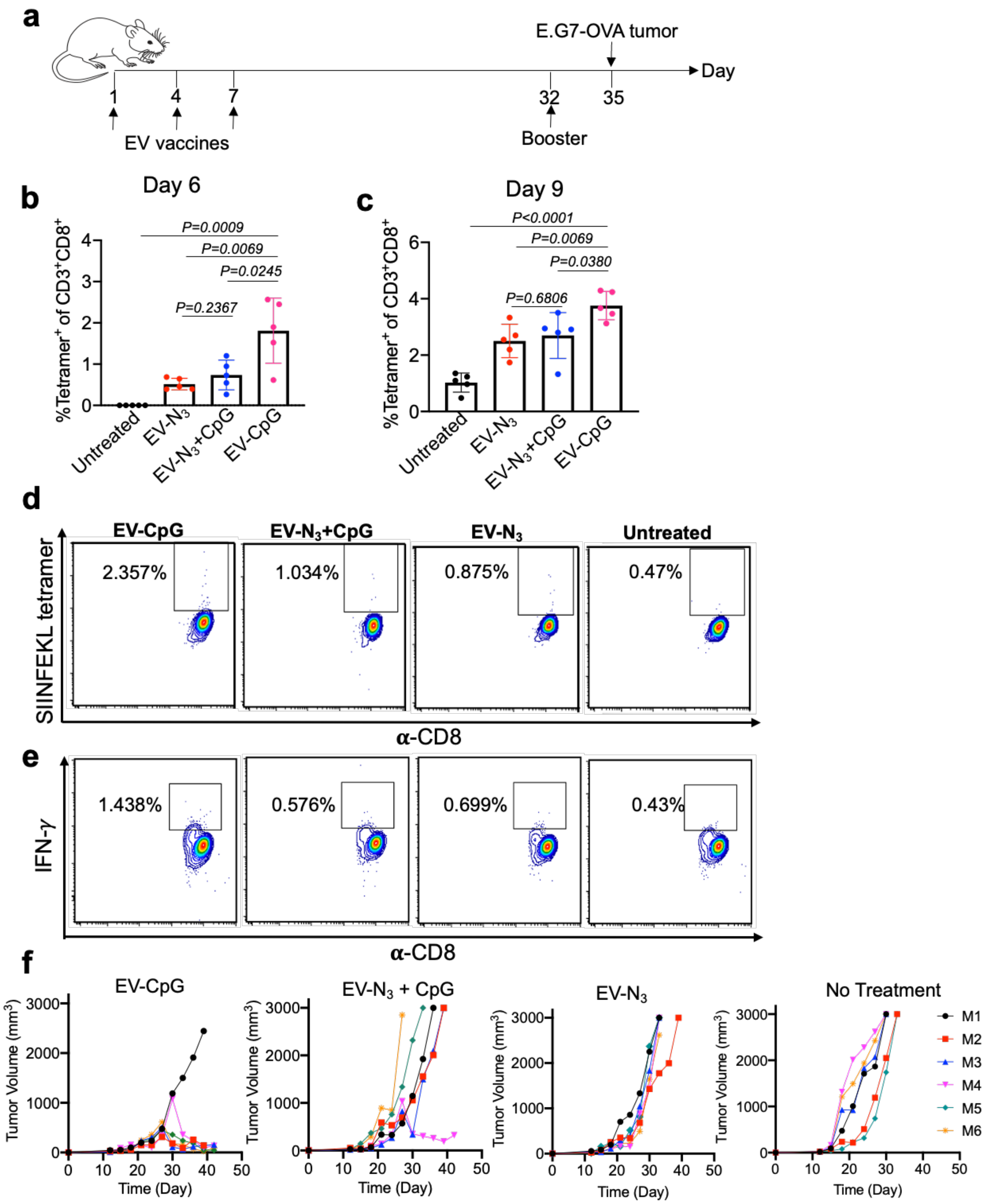


**Figure S12. CpG-conjugated E.G7-OVA exosomes result in enhanced CTL response**. (a) Timeframe of the vaccination study. CpG-conjugated exosomes, the mixture of CpG, exosomes alone, or PBS were subcutaneously injected into C57BL/6 mice on day 1, 4, 7 and 32. E.G7-OVA tumor cells were inoculated on day 35. Shown are the percentage of SIINFEKL tetramer$^+$ cells among CD8$^+$ T cells in PBMC on (b) day 6 and (c) day 9, respectively (n=5). (d) Representative FACS plots of tetramer$^+$ CD8$^+$ T cells in PBMCs on day 20. (e) Representative FACS plots of IFN-$\gamma^+$ CD8$^+$ cells in PBMCs on day 20. (f) E.G7-OVA tumor volume of individual mice for each group over the course of the prophylactic tumor study (n=6). All the numerical data are presented as mean ± SD ($0.01 < {*}P \leq 0.05$; $^{**}P \leq 0.01$; $^{***}P \leq 0.001$; $^{****}P \leq 0.0001$).

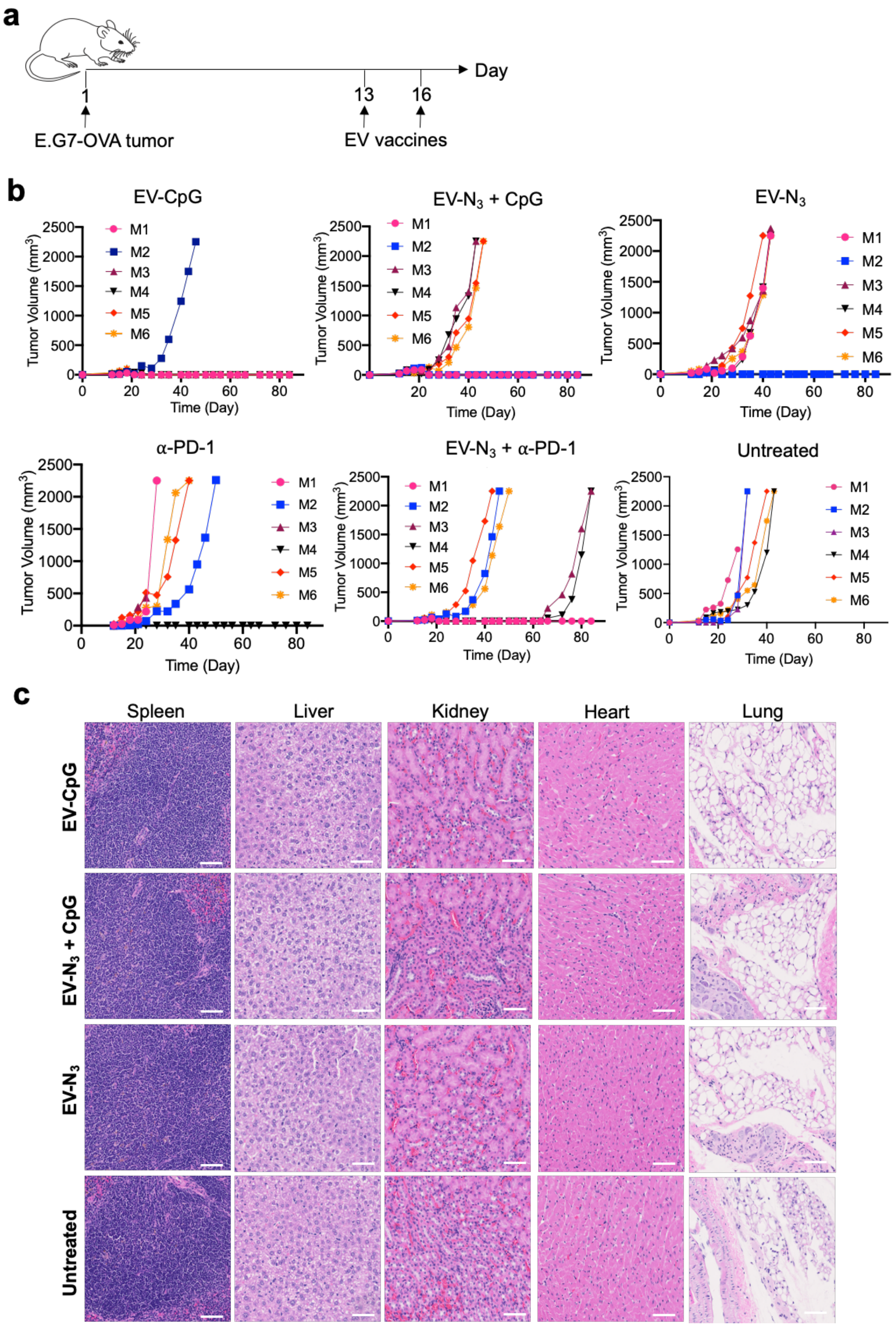


**Figure S13. CpG-conjugated EVs result in improved antitumor efficacy against E.G7-OVA lymphoma.** (a) Timeframe of tumor study. E.G7-OVA tumor was inoculated on day 0. CpG-conjugated EVs, the mixture of CpG and EVs, or EV alone were subcutaneously injected on days 13 and 16. Anti-PD-1 was i.p. administered on days 13 and 16. (b) E.G7-OVA tumor volume of individual mice for each group over the course of the therapeutic tumor study (n=6). (c) Representative H&E stained tissue sections for mice treated with CpG-conjugated EVs, the mixture of EVs and CpG, EV alone, or PBS. Scale bar: 200 μm.

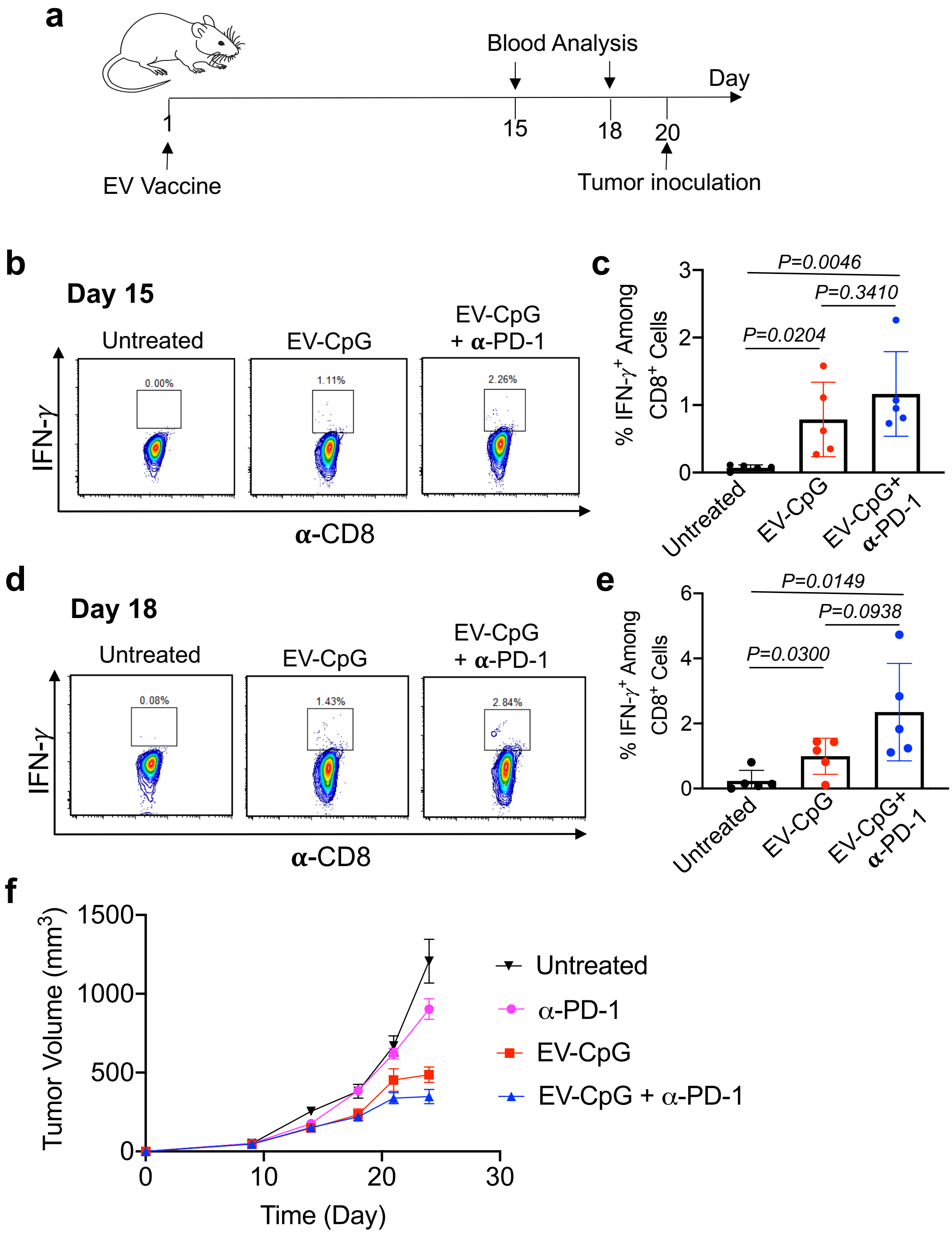


**Figure S14. CpG-conjugated B16F10-derived EVs can induce trp2/gp100-specific CD8$^+$ T cell response**. (a) Timeframe of the vaccination study. C57BL/6 mice (n=5 per group) were subcutaneously injected with EV-CpG or i.p. injected with anti-PD-1 on day 1. PMBCs were isolated on days 15 and 18, followed by *ex vivo* re-stimulation with the mixture of trp2 and gp100 peptides. B16F10 tumor cells were inoculated on day 20. (b) Representative FACS plots of IFN-$\gamma^+$ CD8$^+$ T cells in PBMCs on day 15. (c) Percentage of IFN-$\gamma^+$ cells among CD3$^+$CD8$^+$ T cells in PBMCs on day 15 (n=5). (d) Representative FACS plots of IFN-$\gamma^+$ CD8$^+$ T cells in PBMCs on day 18. (e) Percentage of IFN-$\gamma^+$ cells among CD3$^+$CD8$^+$ T cells in PBMCs on day 18 (n=5). (f) Average B16F10 tumor volume of each group over time (n=5). All the numerical data are presented as mean ± SD except for (f) where data are presented as mean ± SEM ($0.01 < *P \leq 0.05$; $**P \leq 0.01$; $***P \leq 0.001$; $****P \leq 0.0001$).

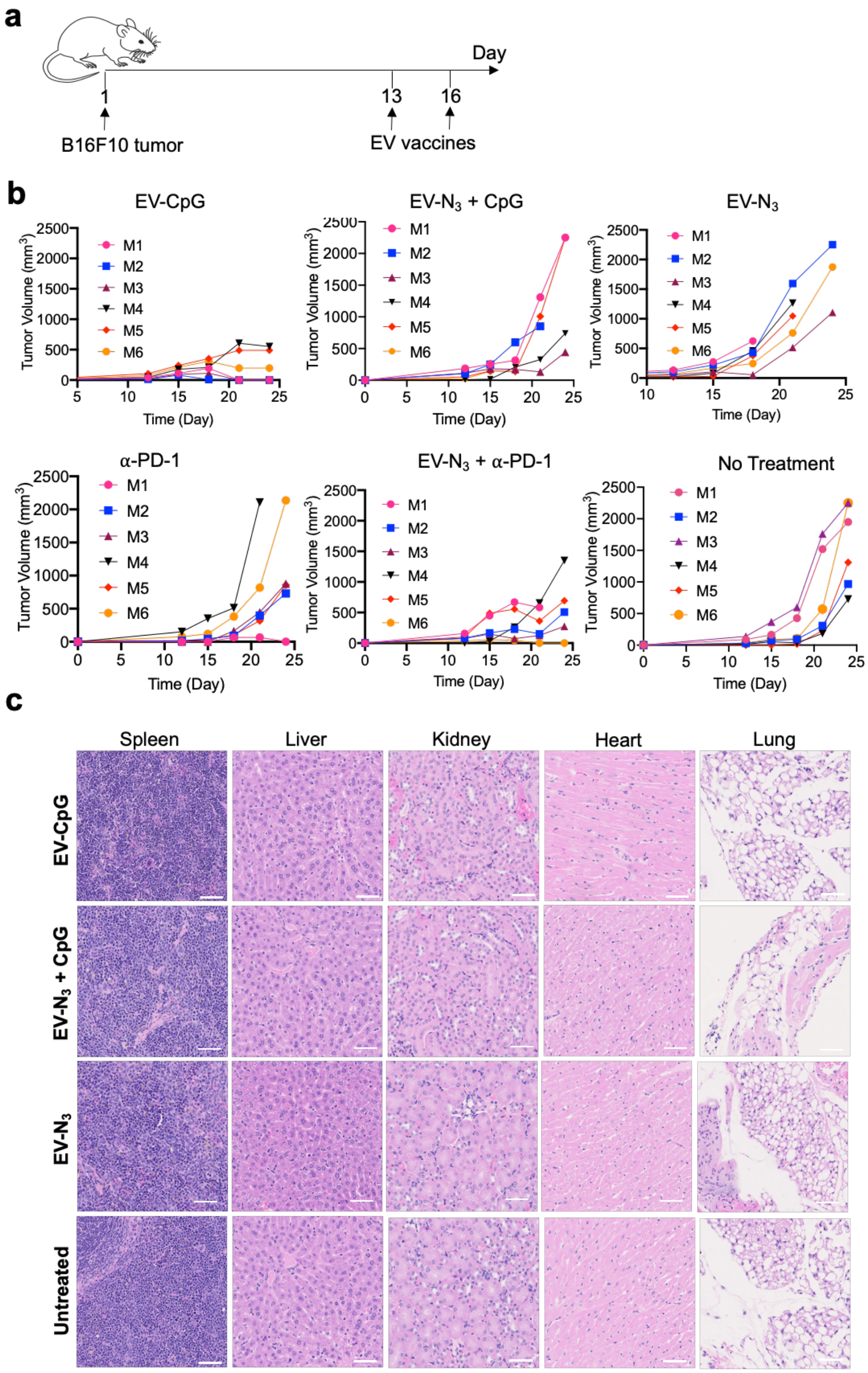


**Figure S15. CpG-conjugated EVs result in improved antitumor efficacy against B16F10 melanoma.** (a) Timeframe of tumor study. B16F10 tumor was inoculated on day 0. CpG-conjugated EVs, the mixture of CpG and EVs, or EV alone were subcutaneously injected on days 13 and 16. Anti-PD-1 was i.p. administered on days 13 and 16. (b) B16F10 tumor volume of individual mice for each group over the course of the therapeutic tumor study (n=6). (c) Representative H&E stained tissue sections for mice with different treatments. Scale bar: 200 μm.

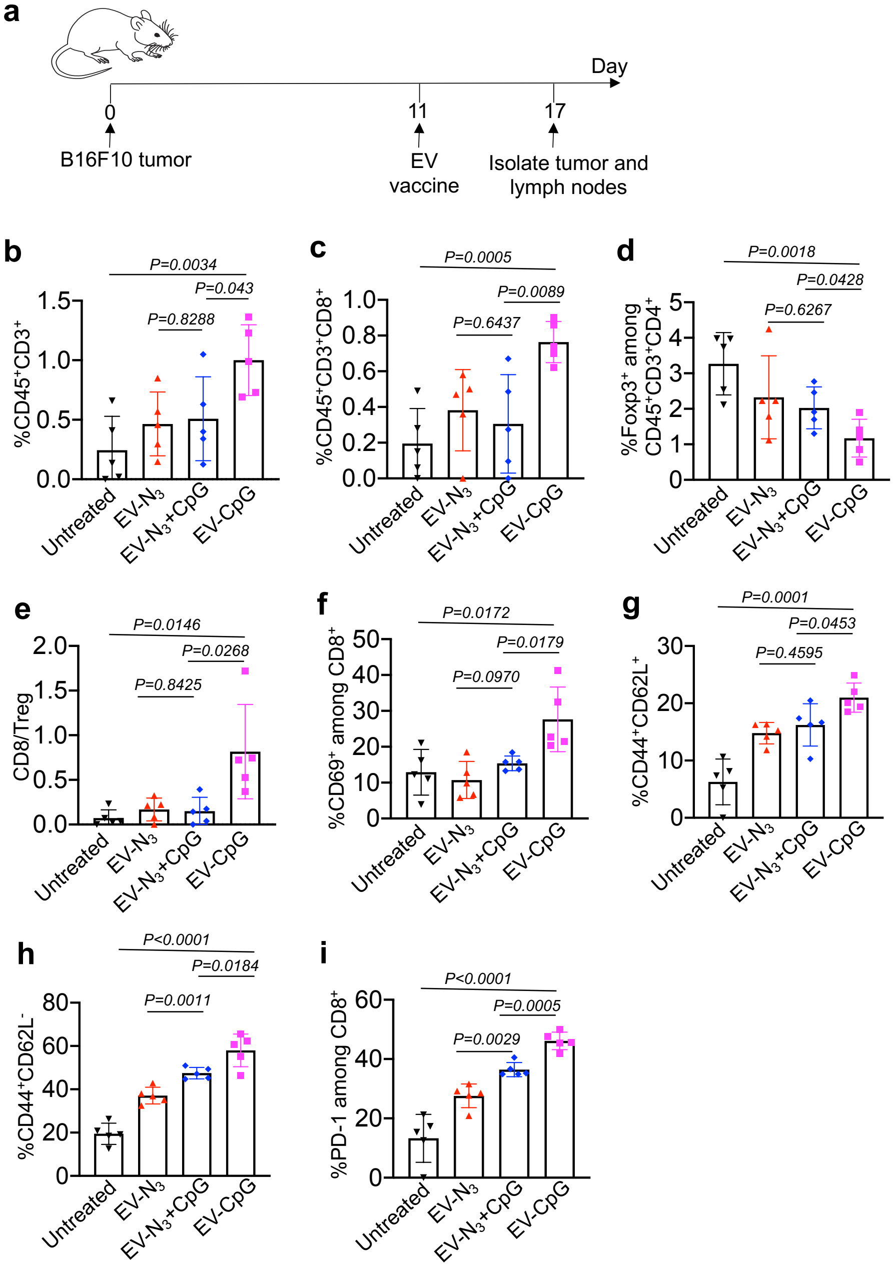


**Figure S16. EV vaccine alters the activation status and phenotype of $CD8^+$ T cells in the tumor microenvironment**. (a) Timeframe of *in vivo* study. B16F10 tumor was subcutaneously inoculated on day 0. CpG-conjugated EVs, the mixture of CpG and EVs, or EV alone (n= 5 per group) were subcutaneously injected on day 11. Tumors were isolated for immune cell analysis on day 17. (b) Percentages of $CD45^+CD3^+$ T cells in tumors. (c) Percentages of $CD8^+$ T cell in tumors. (d) Percentages of $FoxP3^+$ cells among $CD4^+$ T cells in tumors. (e) $CD8^+$ T/Treg number ratios in tumors. (f) Percentages of $CD69^+$ cells among $CD8^+$ T cells in tumors. Also shown are the percentages of (g) central memory T cells ($CD44^+$ $CD62L^+$) and (h) effector memory T cells ($CD44^+$ $CD62L^-$) in tumors. (i) Percentages of PD-$1^+$ cells among $CD8^+$ T cells in tumors. All the numerical data are presented as mean ± SD (0.01 < *$P \leq 0.05$; **$P \leq 0.01$; ***$P \leq 0.001$; ****$P \leq 0.0001$).

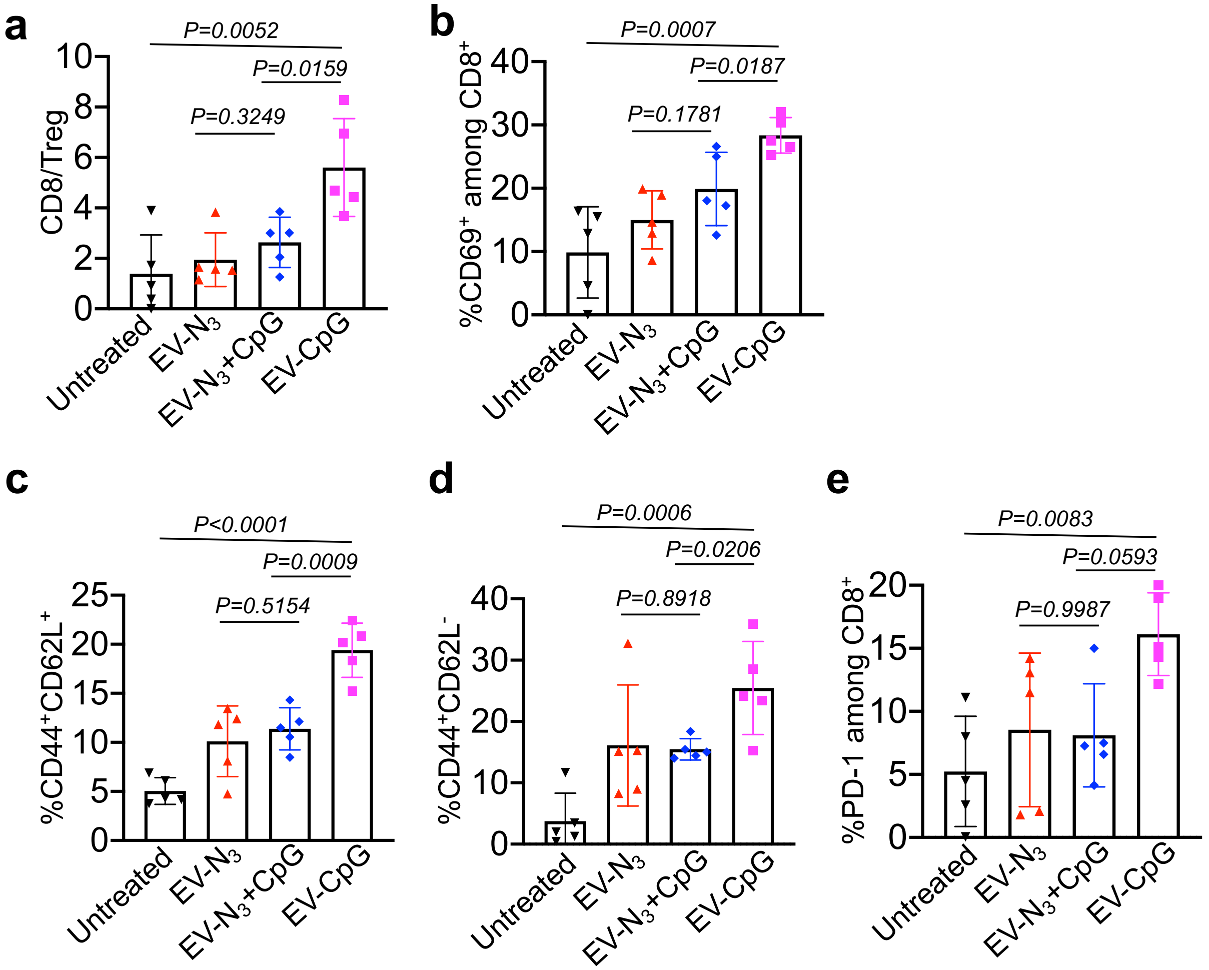


**Figure S17. EV vaccine alters the activation status and phenotype of $CD8^+$ T cells in the tumor-draining lymph nodes**. CpG-conjugated EVs, the mixture of CpG and EVs, or EV alone (n= 5) were subcutaneously injected on day 11. Tumor-draining lymph nodes were isolated for immune cell analysis on day 17. (a) $CD8^+$ T/Treg number ratios in lymph nodes. (b) Percentages of $CD69^+$ cells among $CD8^+$ T cells in lymph nodes. Also shown are the percentages of (c) central memory T cells ($CD44^+CD62L^+$) and (d) effector memory T cells ($CD44^+CD62L^-$) in lymph nodes. (e) Percentages of PD-$1^+$ cells among $CD8^+$ T cells in lymph nodes. All the numerical data are presented as mean ± SD ($0.01 < *P \leq 0.05$; $**P \leq 0.01$; $***P \leq 0.001$; $****P \leq 0.0001$).
