# Supplementary material for: Metabolic tagging of extracellular vesicles and development of enhanced extracellular vesicle based cancer vaccines
Source: Nat Commun. 2023 Dec 5;14:8047. doi: 10.1038/s41467-023-43914-8 (PMC10697976; doi:10.1038/s41467-023-43914-8)
Supplement: Supplementary file 3 — Reporting Summary [file 41467_2023_43914_MOESM3_ESM.pdf]

nature portfolio

Corresponding author(s): Hua Wang
Last updated by author(s): Nov 11, 2023

Reporting Summary

Nature Portfolio wishes to improve the reproducibility of the work that we publish. This form provides structure for consistency and transparency in reporting. For further information on Nature Portfolio policies, see our Editorial Policies and the Editorial Policy Checklist.

Statistics

For all statistical analyses, confirm that the following items are present in the figure legend, table legend, main text, or Methods section.

n/a	Confirmed	
☐	☒	The exact sample size (*n*) for each experimental group/condition, given as a discrete number and unit of measurement
☐	☒	A statement on whether measurements were taken from distinct samples or whether the same sample was measured repeatedly
☐	☒	The statistical test(s) used AND whether they are one- or two-sided<br>*Only common tests should be described solely by name; describe more complex techniques in the Methods section.*
☐	☒	A description of all covariates tested
☐	☒	A description of any assumptions or corrections, such as tests of normality and adjustment for multiple comparisons
☐	☒	A full description of the statistical parameters including central tendency (e.g. means) or other basic estimates (e.g. regression coefficient) AND variation (e.g. standard deviation) or associated estimates of uncertainty (e.g. confidence intervals)
☐	☒	For null hypothesis testing, the test statistic (e.g. *F*, *t*, *r*) with confidence intervals, effect sizes, degrees of freedom and *P* value noted<br>*Give P values as exact values whenever suitable.*
☒	☐	For Bayesian analysis, information on the choice of priors and Markov chain Monte Carlo settings
☒	☐	For hierarchical and complex designs, identification of the appropriate level for tests and full reporting of outcomes
☒	☐	Estimates of effect sizes (e.g. Cohen's *d*, Pearson's *r*), indicating how they were calculated

Our web collection on statistics for biologists contains articles on many of the points above.

Software and code

Policy information about availability of computer code

Data collection	No computer code was used. Routine softwares for flow cytometry and figure plotting are described in the manuscript and reported below.
Data analysis	No computer code was used. Routine softwares for flow cytometry and figure plotting are described in the manuscript and reported below.

For manuscripts utilizing custom algorithms or software that are central to the research but not yet described in published literature, software must be made available to editors and reviewers. We strongly encourage code deposition in a community repository (e.g. GitHub). See the Nature Portfolio guidelines for submitting code & software for further information.

Data

Policy information about availability of data

All manuscripts must include a data availability statement. This statement should provide the following information, where applicable:

- Accession codes, unique identifiers, or web links for publicly available datasets
- A description of any restrictions on data availability
- For clinical datasets or third party data, please ensure that the statement adheres to our policy

All data provided in this study can be found in the main text, figures, supplementary information, and source data files.

Research involving human participants, their data, or biological material

Policy information about studies with human participants or human data. See also policy information about sex, gender (identity/presentation), and sexual orientation and race, ethnicity and racism.

Reporting on sex and gender	N/A
Reporting on race, ethnicity, or other socially relevant groupings	N/A
Population characteristics	N/A
Recruitment	N/A
Ethics oversight	N/A

Note that full information on the approval of the study protocol must also be provided in the manuscript.

Field-specific reporting

Please select the one below that is the best fit for your research. If you are not sure, read the appropriate sections before making your selection.

- [x] Life sciences
- [ ] Behavioural & social sciences
- [ ] Ecological, evolutionary & environmental sciences

For a reference copy of the document with all sections, see nature.com/documents/nr-reporting-summary-flat.pdf

Life sciences study design

All studies must disclose on these points even when the disclosure is negative.

Sample size	n=4-8 for in vitro studies, n=5-10 for animal studies, sample size was given for each specific experiment in the Methods section and Figure captions.
Data exclusions	No data were excluded.
Replication	All studies have been repeated at least once.
Randomization	Cells and animals were randomly allocated to different groups before treatment.
Blinding	The investigators were aware of the allocated groups for research needs.

Reporting for specific materials, systems and methods

We require information from authors about some types of materials, experimental systems and methods used in many studies. Here, indicate whether each material, system or method listed is relevant to your study. If you are not sure if a list item applies to your research, read the appropriate section before selecting a response.

Materials & experimental systems

n/a	Involved in the study	
[ ]	[x]	Antibodies
[ ]	[x]	Eukaryotic cell lines
[x]	[ ]	Palaeontology and archaeology
[ ]	[x]	Animals and other organisms
[x]	[ ]	Clinical data
[x]	[ ]	Dual use research of concern
[x]	[ ]	Plants

Methods

n/a	Involved in the study	
[x]	[ ]	ChIP-seq
[ ]	[x]	Flow cytometry
[x]	[ ]	MRI-based neuroimaging

Antibodies

Antibodies used	Primary antibodies used in this study, including PE-conjugated anti-CD11b (Invitrogen), PE/Cy7-conjugated anti-CD11c (Invitrogen), APC-conjugated anti-CD86 (Invitrogen), PE/Cy5.5- conjugated anti-CD3-ε (Invitrogen), Alexa Fluor 700-conjugated anti-CD8-α (Invitrogen), PE/Cy7-conjugated anti-PD-1 (Invitrogen), PE-conjugated anti-CTLA-4 (Invitrogen), APC-conjugated anti-LAG-3 (Invitrogen), and FITC-conjugated anti-MHCII (Invitrogen) were purchased from Thermo Fisher Scientific (Waltham, MA, USA).

Validation	All validation files can be found in the manufacturer's websites.

Eukaryotic cell lines

Policy information about cell lines and Sex and Gender in Research

Cell line source(s)	E.G7-OVA (ATCC), B16-F10 (ATCC), 4T1 (ATCC), LS174T (ATCC), GL261 (ATCC), and BxPC-3 (ATCC)
Authentication	Authentication steps were performed by ATCC.
Mycoplasma contamination	All cell lines are negative for Mycoplasma contamination
Commonly misidentified lines (See ICLAC register)	No commonly misidentified lines were used in the studies.

Animals and other research organisms

Policy information about studies involving animals; ARRIVE guidelines recommended for reporting animal research, and Sex and Gender in Research

Laboratory animals	C57BL/6 (5-7 weeks)
Wild animals	No wild animals were used in this study
Reporting on sex	Only Female mice were used in this study, due to the challenge in housing a large number of male mice.
Field-collected samples	No Field=collected samples were used in this study
Ethics oversight	All procedures involving animals were done in compliance with National Institutes of Health and Institutional guidelines with approval from the Institutional Animal Care and Use Committee at the University of Illinois at Urbana-Champaign.

Note that full information on the approval of the study protocol must also be provided in the manuscript.

Plants

Seed stocks	N/A
Novel plant genotypes	N/A
Authentication	N/A

Flow Cytometry

Plots

Confirm that:

- [x] The axis labels state the marker and fluorochrome used (e.g. CD4-FITC).
- [x] The axis scales are clearly visible. Include numbers along axes only for bottom left plot of group (a 'group' is an analysis of identical markers).
- [x] All plots are contour plots with outliers or pseudocolor plots.
- [x] A numerical value for number of cells or percentage (with statistics) is provided.

Methodology

Sample preparation	Treated cells were washed and stained with fluorophore-conjugated antibodies, washed for three times, and stored in 0.4% PFA in FACS buffer, prior to flow cytometry run.
Instrument	Attune NxT flow cytometer
Software	FCS Express v6 and v7

Cell population abundance	In all experiments, at least 10,000 cells were counted and analyzed.
Gating strategy	Details provided in the manuscript. In general, FSC-SSC, singlets, and live cells were pre-gated.

☒ Tick this box to confirm that a figure exemplifying the gating strategy is provided in the Supplementary Information.
